# Evaluation of Neuro Images for the Diagnosis of Alzheimer's Disease Using Deep Learning Neural Network

**DOI:** 10.3389/fpubh.2022.834032

**Published:** 2022-02-07

**Authors:** Ahila A, Poongodi M, Mounir Hamdi, Sami Bourouis, Kulhanek Rastislav, Faizaan Mohmed

**Affiliations:** ^1^Department of Electronics and Communication Engineering, Sethu Institute of Technology, Kariapatti, India; ^2^College of Science and Engineering, Hamad Bin Khalifa University, Qatar Foundation, Doha, Qatar; ^3^Department of Information Technology, College of Computers and Information Technology, Taif University, Taif, Saudi Arabia; ^4^Information Systems Department, Faculty of Management, Comenius University in Bratislava, Bratislava, Slovakia; ^5^School of Creative Tech, University of Bolton, Bolton, United Kingdom

**Keywords:** Alzheimer's disease, accuracy, convolutional neural network, deep learning, feature extraction, image analysis, image classification and positron emission tomography

## Abstract

Alzheimer's Disease (AD) is a progressive, neurodegenerative brain disease and is an incurable ailment. No drug exists for AD, but its progression can be delayed if the disorder is identified at its initial stage. Therefore, an early analysis of AD is of fundamental importance for patient care and efficient treatment. Neuroimaging techniques aim to assist the physician in the diagnosis of brain disorders by using images. Positron emission tomography (PET) is a kind of neuroimaging technique employed to create 3D images of the brain. Due to many PET images, researchers attempted to develop computer-aided diagnosis (CAD) to differentiate normal control from AD. Most of the earlier methods used image processing techniques for preprocessing and attributes extraction and then developed a model or classifier to classify the brain images. As a result, the retrieved features had a significant impact on the recognition rate of previous techniques. A novel and enhanced CAD system based on a convolutional neural network (CNN) is formulated to address this issue, capable of discriminating normal control from Alzheimer's disease patients. The proposed approach is evaluated using the 18FDG-PET images of 855 patients, including 635 normal control and 220 Alzheimer's disease patients from the ADNI database. The result showed that the proposed CAD system yields an accuracy of 96%, a sensitivity of 96%, and a specificity of 94%, leading to splendid performance when related to the methods already in use that are specified in the literature.

## 1. Introduction

Alzheimer's disease (AD) is a type of brain disease which regularly affects people over 65 years old. It is a progressive and neurodegenerative disorder, meaning that, it becomes worse with time. According to an Alzheimer's Association report, around 55 million people are living with AD worldwide and it is envisioned that the number of AD patients will reach 152 million by 2,050. The number of people with AD is progressively increasing worldwide. The prime objective of this paper is to develop a robust classification system for AD diagnosis using a convolutional neural network (CNN). In this approach, the 3D image classification problem is completely solved by converting them into 2D images. The proposed system uses whole brain images for AD diagnosis instead of region of interest. Thus, there is no need for a segmentation algorithm. Furthermore, the proposed system uses CNN for classification. CNN learns the features from images. Pre-processing like noise removal, enhancement, and feature extraction are not needed. AD starts with impairment of memory functions followed by cognitive functions with behavioral impairments (1). Some common symptoms of AD are: memory loss, a lack of initiative, difficulty in expressing thoughts and recognizing people or relations, personality changes, and poor judgement, etc. AD is an incurable brain disorder. Currently, no effective treatment has yet been fully discovered, but it is possible to slow down the progression of AD if the disorder is identified at its initial stage. Therefore, it is of primary significance to identify AD at an early stage for patient care, as well as effective treatment. Furthermore, if the condition is diagnosed early on, some of the changes induced by AD can be reversed, allowing patients to keep their everyday lives. However, diagnosis of AD still remains a highly complicated task, especially at an early stage while the disorder offers greater opportunities to be treated (2).

Over the past few years, several neuro imaging modalities which include magnetic resonance imaging (MRI), positron emission tomography (PET) (3), and single-photon emission computerized tomography (SPECT) have been confirmed to be very powerful within the diagnosis of AD. PET is a non-invasive neuro imaging technique that uses an imaging agent like 18FDG-PET to monitor the brain's glucose intake. Brain activity is related to glucose consumption. During PET scanning, a position emitting radionuclide tracer with 18FDG is delivered in the body. The glucose uptake shows tissue enzymatic reactions when the quantities of these traces are scanned with a camera. In seriously damaged AD patients, particular brain areas showed reduced glucose intake, together with bilateral areas inside the temporal and partial lobes, posterior cingulate, frontal lobes, and entire brain (4). Research studies proved that FDG-PET is a good candidate for AD diagnosis and can be used to assist the physicians or an expert to analyze and diagnosis AD in early stages. Consequently, this paper proposes an automated computer-aided design (CAD) system for the diagnosis of AD using 18FDG-PET images. The foremost awareness of this research work is to distinguish AD patients from normal control (NC) employing CNN (5).

Classification of medical images via visual examination by an expert or a physician can be subjective and prone to errors. As a result, researchers attempted to create an automatic CAD system that could distinguish AD from NC participants using picture data attributes. Over the past few years, numerous machine learning models have evolved and are being used for examining neuro reading that allows researchers to capture the structure or functional changes related to AD including support vector machine (SVM) (6) classification in SPECT and learning vector quantization-SVM (LVQ-SVM) classification in structural MRI (7).

Wysoczánski et al. (1) investigated the strength of random forest (RF) for diagnosing AD. The authors used partial least square (PLS) for extracting features from the image. This method is tested on SPECT images downloaded from the ADNI dataset. A new CAD system for differentiating AD from NC based on PET image features was developed by Cabral and, Silveria (4). Garali et al. (8) proposed a classification system using favorite class ensembles for differentiating AD and MCI from NC using PET images. Each classifier in the ensemble uses a different set of brain voxels. Two base classifiers SVM and random forest are employed to create the ensemble classifier. Results showed that this ensemble classifier outperforms the corresponding single classifier. However, higher computational cost is the drawback of this system. Markiewicz et al. (9) presented a region-based method to classify AD from NC using 16 anatomical regions of interest (ROIs), and the first four times, as well as their entropy, are computed and used as feature vectors. The capacity of ROIs to discriminate PET images is then ranked using ROC curves. Finally, 21 capabilities are selected and fed as an input to both SVM and RF classifiers. Results proved that this method achieved high classification accuracy using 166 anatomical ROIs when compared to other methods. Poongodi and Bose (10) proposed a deep learning model for AD diagnosis from MRI images. A deep neural network is designed with a convolution layer, normalization layer, and pooling layer. The effectiveness of this method is validated on the OASIS dataset. Results showed improved performance of this particular method when compared to other methods. A boosting classifier to classify PET images was developed by Poongodi and Bose (11). It is the combination of simple classifiers, which performs segmentation, feature extraction, and feature selection in order to make the input image fit for the classification task. This classifier was validated on the ADNI dataset and achieved an accuracy of 90.97%.

## 2. Distinct Features of the Proposed Method

In the literature, researchers have developed an automated CAD system using various soft computing models (12). Most of the researchers have used either full brain images or set features for diagnosing AD. Each method has its own merits and demerits. However, none of the methods offer a consistent outcome. Keeping this in mind, this research work intends to design and render a CAD system for discriminating AD from NC with the help of CNN. In the proposed CAD system, the first 15 slices and last 15 slices are discarded to remove unwanted regions. Dimension-reduced 18FDG-PET images are fed as input into the deep neural network. CNN is used for classifying FDG-PET snapshots into NC and AD. The efficacy of CNN is evaluated by measuring accuracy, sensitivity, and specificity, and is compared with the existing methods (13–16).

For solving the shortcomings of the methods elaborated in the literature, this paper presents a novel computer diagnosis system using a deep convolutional neural network. The conventional neural network has the potential of recognizing images with minimal processing. The novel features of the proposed system are: 3D images are transformed into 2D images so as to reduce the computational time, 2D images are further grouped at specific intervals for reducing the dimension, image segmentation is not required in this proposed approach, and no image processing techniques are needed to extract features from the images.

From the existing literature survey, it could be seen that, in existing computer-aided diagnosis systems, in those intended to classify the brain images (5), it was observed that most of the systems employ image processing methods to classify the data, therefore, a machine learning model was designed to obtain the features. Also, few methods use only the information from regions of interest (17). However, these methods require increased computational time. In addition, the classification accuracy depends mainly upon the selected features. Researchers developed hybrid models by combining a deep learning neural network and machine learning model to increase the classification accuracy, but these models required increased memory usage and increased time (18).

To overcome these issues, the proposed CAD system employs the consideration of entire brain images instead of regions of interest. The 3D picture type problem is completely solved with the aid of converting them into 2D images. Venugopalan et al. (19) proposed a set of rules that might help in robot surgical treatment. To run on such soft tissues, software program-driven techniques and algorithms have to be extra particular in choosing the highest quality direction for attaining the procedural region. Statistical analysis has determined whether the proposed approach might be outperforming under the favorable learning rate, discount factor, and the exploration factor (20). The network is built with multiple layers to learn features through a training process, which eliminates the need for extracting the features, resulting in higher prediction performance when compared to other approaches. Reddy et al. (21) focused on classifying tomato disease with a machine learning model used to predict agricultural disaster. The features were extracted from the dataset using the hybrid-predominant element evaluation-whale optimization algorithm and the extracted features were fed into a deep neural network for classification of tomato diseases. Srinivasu et al. (22) focused on improving quality, a preprocessing technique was implemented in a multimodal stroke dataset from the Kaggle repository. To achieve homogeneity, a label encoder technique was used and dataset missing values were replaced with attribute means. Resampling techniques were used to balance the dataset and to obtain accurate results. The following is how the rest of the paper is organized: The demographic details of the dataset utilized in this work are presented in section 2. Section 3 formulates the unique features of the proposed CAD device for AD prognosis. Section 4 presents the unique evaluation of numerical results and evaluation of the results. Finally, section 5 concludes the paper with few suggestions toward future research in closer vicinity of this study.

## 3. Dataset Detail

The neuroimaging dataset that was used in this study is presented in this section.

The visual dataset used in this study was acquired from ADNI, which is publicly available (http://adni.loni.usc.edu/methods/pet-analysis-methods). The ADNI database was released in 2003 by the NIA, NIBIB, and FDA. The ADNI was designed for the diagnosis of AD at the earliest stage and tracking of progression of AD with several biomarkers such as MRI, PET, and SPECT. ADNI helps researchers to gather, collect, and use the image data including PET to measure and track the progression of AD.

18FDG-PET images of 855 patients and their final diagnosis were collected from ADNI. The detailed procedure for 18FDG-PET scanning can be found on the website (http://adni.loni.usc.edu/methods/pet-analysis-methods). The total data have 855 samples including 635 NC and 220 AD subjects. The dataset is split into two categories: training data and validation data. Around 90% of the data, referred to as training data, was used to construct the model, while the remaining 10%, referred to as validation data, was used to validate the model. Description of 18FDG-PET images investigated in this work is provided in Table 1. Only subjects with CDR of 0.5 or 1 for AD and 0 for NC were chosen. Classification was based on MMSE (Mini Mental State Examination) scores of 24 to 30 for NC and 22 to 26 for AD. The resulting volumes of images have been represented by means of a matrix of size 256x256x96 yielding a total of 6291456 voxels. The values are denoted as mean plus or minus std deviation.

**Table 1:** Demographic details of 18FDG-PET image dataset.

**Factors**	**Normal control**	**Alzheimer's disease**
Number of subjects	635	220
Age	70	75
Gender (M/F)	35/15	30/20
MMSE	24–30	20–26
CDR	0	1.5 or 1

## 4. Proposed CAD System

### 4.1. In-linestyle

The capture of 18FDG-PET images in ADNI followed a conventional methodology. The pictures were co-registered, averaged, aligned, normalized, and smoothed to a similar resolution of 8 mm FWHM 30–60 min after injection. Each image was examined for artifacts and, if needed, its orientation was changed.

**Figure 1:**
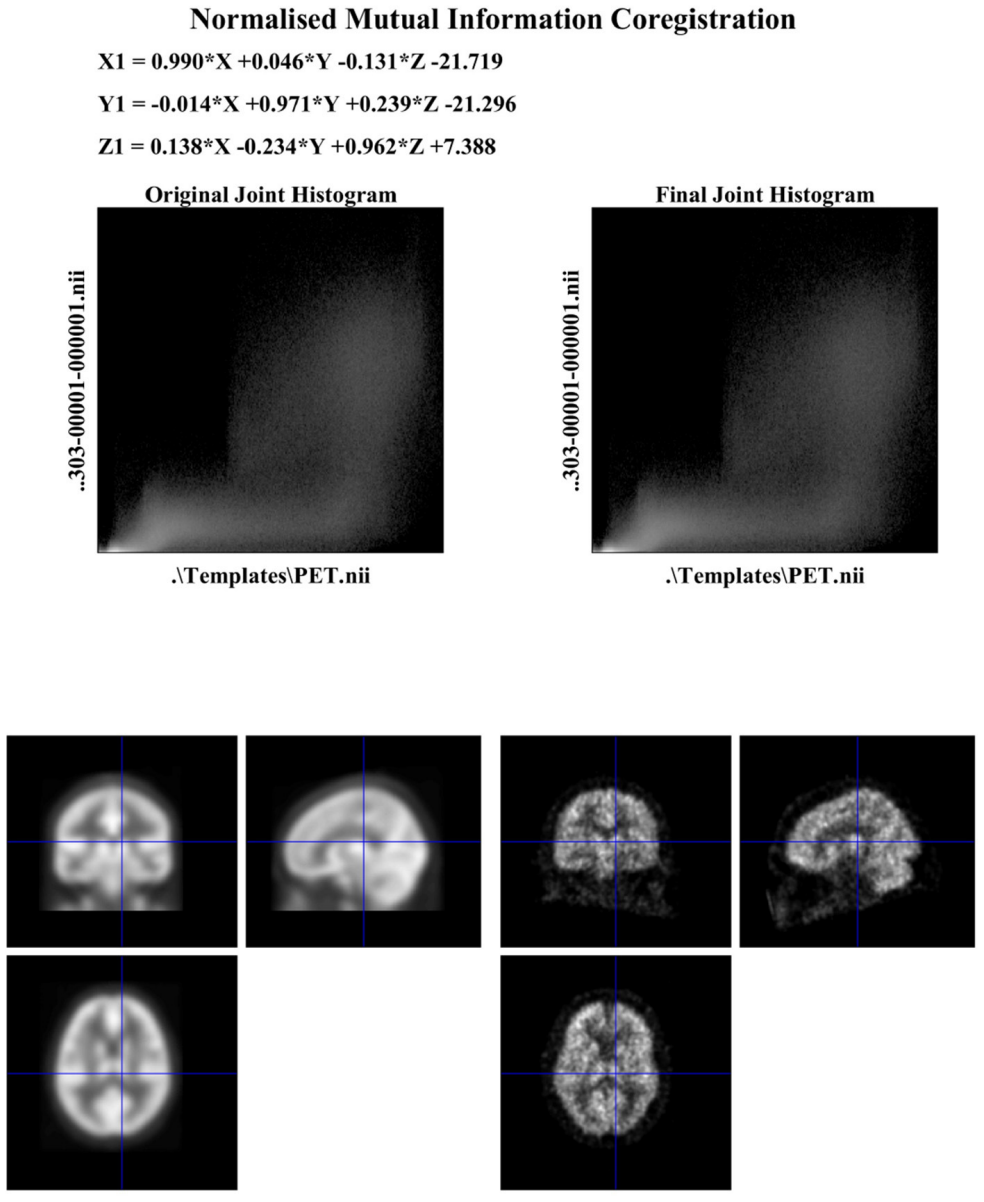
Outcome of co-registration.

Subsequently, the FDG-PET images have been normalized through an affine model with 12 parameters using SPM12 software, normalized in intensity, and converted to a uniform isotropic resolution of 8 mm FWHM. All the images were reduced to a size of 160 x 160 x 96 for analysis. Figure 1 shows the sample of co-registration using SPM12.

### 4.2. Classification

**Figure 2:**
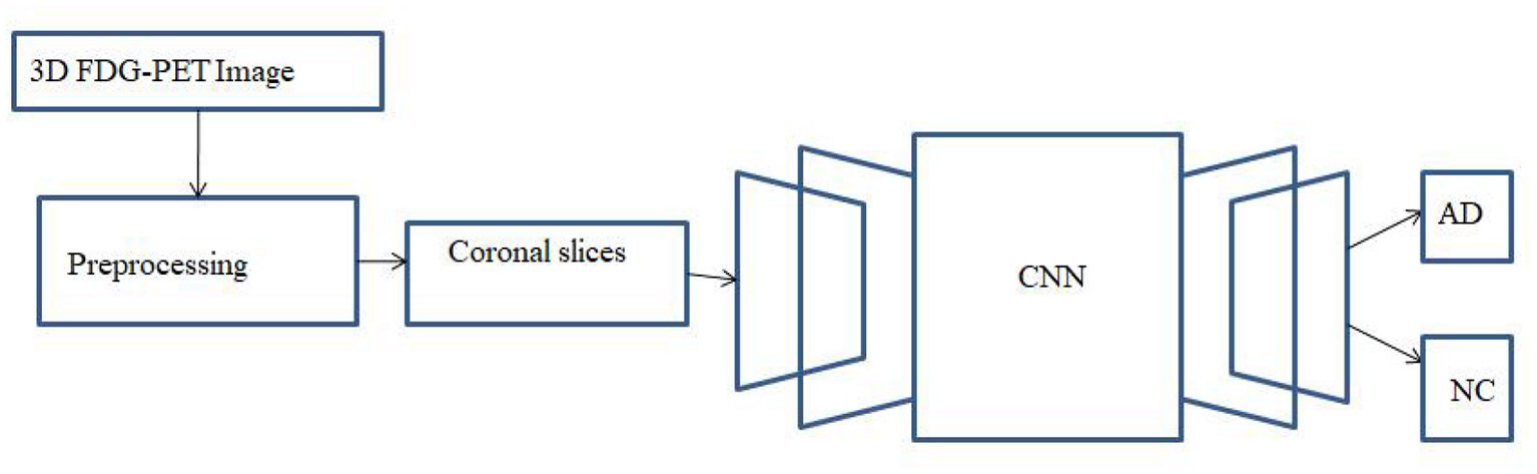
Framework of the proposed CAD system.

Numerous methods have been proposed for AD diagnosis using various approaches like RF, SVM, and ANN. Each method has its own characteristics. In recent years, deep learning neural networks were used for medical photo evaluation and computer vision problems due to its ability to capture the features from 2D images. Additionally, CNN has the potential to recognize visual patterns with minimal preprocessing. CNN is robust to spatial and geometric transformations. In this paper, a 2D CNN is built to differentiate AD from NC. Figure 2 depicts the suggested CAD system's framework. Each 3D FDG-PET image is transformed into multiple 2D images along the coronal (axial) direction, as shown in Figure 2. The initial 15 and closing 15 slices are discarded to do away with skull and other undesirable regions. Subsequently, the 2D slices are arranged into groups at particular intervals with some overlaps and averaged to reduce the dimension and computational overhead. CNN is developed with many layers and trained to capture useful features. The features generated by convolution layers are implemented to absolutely connected layers followed by means of a smooth max to make the final decision.

**Figure 3:**
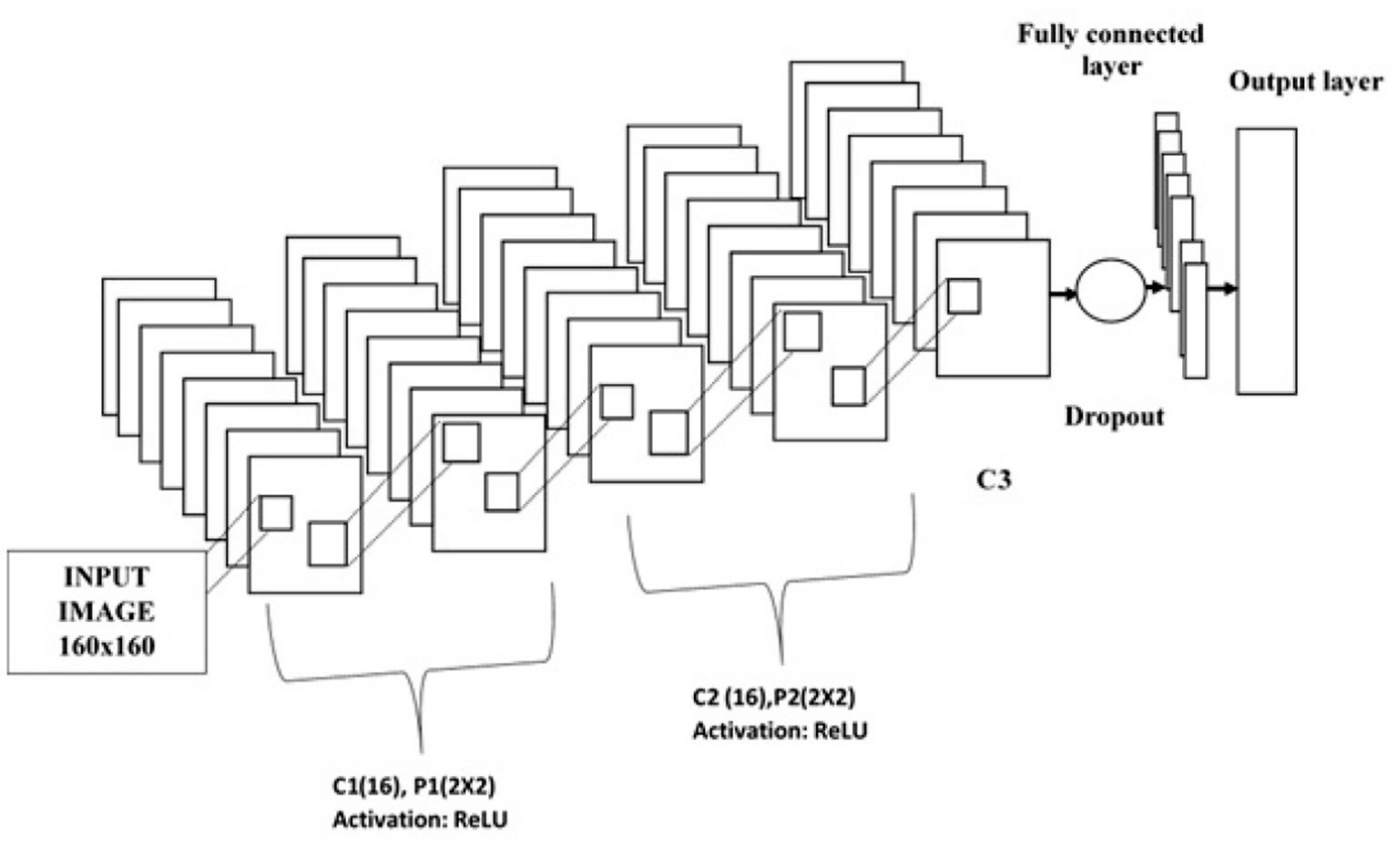
Convolutional neural network.

The convolution layer, pooling or subsampling layer, fully connected layer, and soft max classification layer are the layers that make up a CNN. The designed 2D CNN is composed of an input layer, three convolutional layers, two subsampling or pooling layers, a drop out layer, a fully connected layer, and a smooth max layer as shown in Figure 3. Parameters employed for constructing the CNN are tabulated in Table 2. The hyper parameters are tuned by experimentation. After many trails, the values are fixed. The designed CNN has an input layer size of 160 x 160. The sizes of receptive fields or filters for convolutional layers are set to 3 x 3 and kernel numbers are 8, 16, and 32 for C1, C2, and C3, respectively. Convolutional layers are used for extracting features from the image. The first C1 generates low level features with a size of 158 x 158. The pooling layer is used to reduce the dimension of the features. Max pooling function is adopted. Subsequent to P1, these features are reduced into 79 x 79. Then, C2 generates mid-level features with a size of 39 x 39 and these features are down-sampled into 20 x 20. Finally, C3 generates high-level features with a size of 10 x 10. These features are concatenated and taken as the feature vector. The obtained feature vector is fed to FC followed by the soft max layer. In this work, the rectified linear unit (ReLU) activation function is adopted to introduce non-linearity. SGDM is used to train the network by minimizing the cross entropy loss, and maximum iteration is set to 500. The drop out layer is utilized to improve the generalization ability and to prevent the overfitting problem.

**Table 2:** CNN parameters employed.

**Sl.No**.	**Layer**	**Kernel size/stride**	**No. of kernels**	**Size**
1	Input image			160 x 160 x 1
2	Convolution layer 1 (C1)	3 x 3/1	16	158 x 158 x 16
3	Pooling layer 1 (P1)	2 x 2/2		79 x 79 x 16
4	Convolution layer 2 (C2)	3 x 3/1	32	39 x 39 x 32
5	Pooling layer 2 (P2)	2 x 2/2		20 x 20 x 32
6	Convolution layer 3 (C3)	3 x 3/2	64	10 x 10 x 64
7	Flatten			6,400
8	Fully connected layer	512		512
9	Soft max	2		2

## 5. Numerical Results and Discussion

This section depicts the numerical results of the proposed CAD system for AD diagnosis from FDG-PET images. It also goes through the simulation environment and performance measures that were used to compare the proposed system to other existing approaches.

### 5.1. Simulation Platform

The main objective of simulation platform is to provide better results for visualization and interpretation. The developed CAD system is implemented on the MATLAB 2018a platform using the deep learning toolbox and executed in an Intel core i5 processor with 2.5 GHz speed and 12 GB of RAM.

### 5.2. Performance Indicators

To estimate the efficacy of the proposed CAD system, this paper evaluates some widely used performance indicators, such as confusion matrix, region of curve (ROC), classification accuracy, sensitivity, and specificity. Accuracy calculates the proportion between true positive (correctly classified data) and total data, and shall be mathematically represented as given in Equation (1).

Specificity is the percentage of normal control patients who are identified as not having AD, and can be expressed as mentioned in Equation (2).


(1) $$\begin{array}{l} \begin{array}{c} \text{Accuracy}=\frac{TP+TN}{TP+TN+FP+FN} \end{array} \end{array}$$

(2) $$\begin{array}{l} \begin{array}{c} \text{Specificity}=\frac{TN}{TN+FP} \end{array} \end{array}$$

Sensitivity computes the percentage of AD patients who are diagnosed and as such sensitivity is calculated as expressed in Equation (3).


(3) $$\begin{array}{l} \begin{array}{c} \text{Sensitivity}=\frac{TP}{TP+FN} \end{array} \end{array}$$

True positive is represented by TP, true negative is represented by TN, false negative is represented by FN, and false positive is represented by FP.

### 5.3. Experimental Treatment

In this work, an automated CAD system for the diagnosis of brain disorders is developed and implemented accordingly. The proposed system was employed to differentiate AD patients from NC. The FDG-PET image dataset was utilized in the simulation and was gathered from the ADNI database. The FDG-PET images were acquired from 855 patients including 635 NC and 220 AD patients.

**Figure 4:**
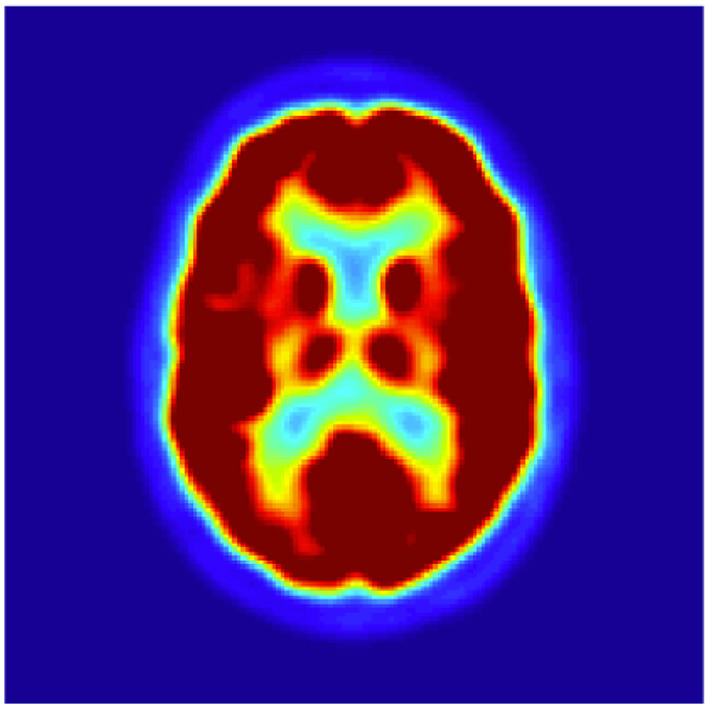
Sample of preprocessed NC images.

**Figure 5:**
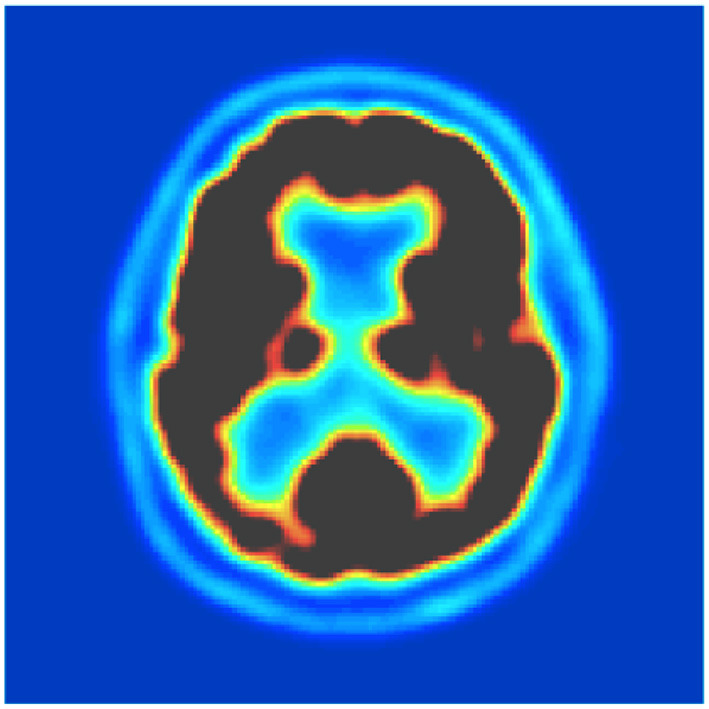
Sample of preprocessed AD images.

**Figure 6:**
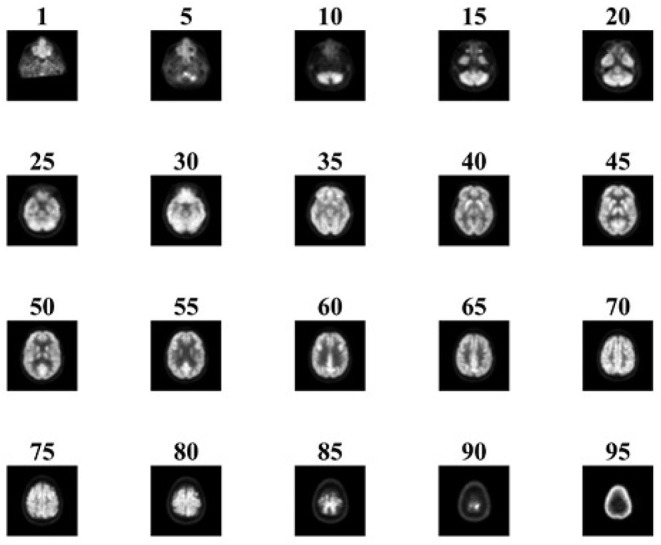
Sample slices of NC patient.

**Figure 7:**
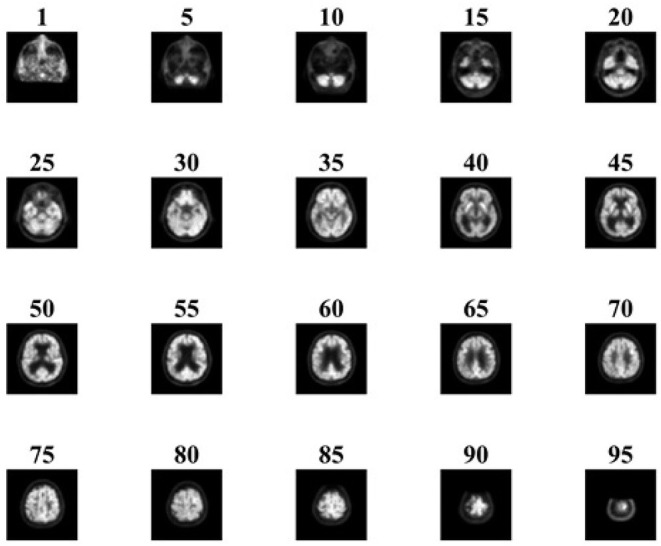
Sample slices of AD patient.

The collected images are preprocessed using SPM12. The samples of preprocessed images of NC and AD patients are given in Figures 4, 5, respectively. It is obvious that hypometabolism is visible in AD patients. Moreover, the first 15 and last 15 slices have redundant information. Slices of these regions of AD and NC patients are demonstrated in Figures 6, 7, respectively. These slices are discarded to remove unwanted regions and make the image fit for further processing. Preprocessed and dimension-reduced images are fed as input into the designed system. CNNs are a kind of deep learning neural network that have proven to be very effective in image classification recognition problems. The CNN learns features from the images acquired using its own convolutional layer. Additionally, CNNs can recognize patterns with extreme variability and some geometric transformations such as scaling, rotation, translation, and noise.

**Figure 8:**
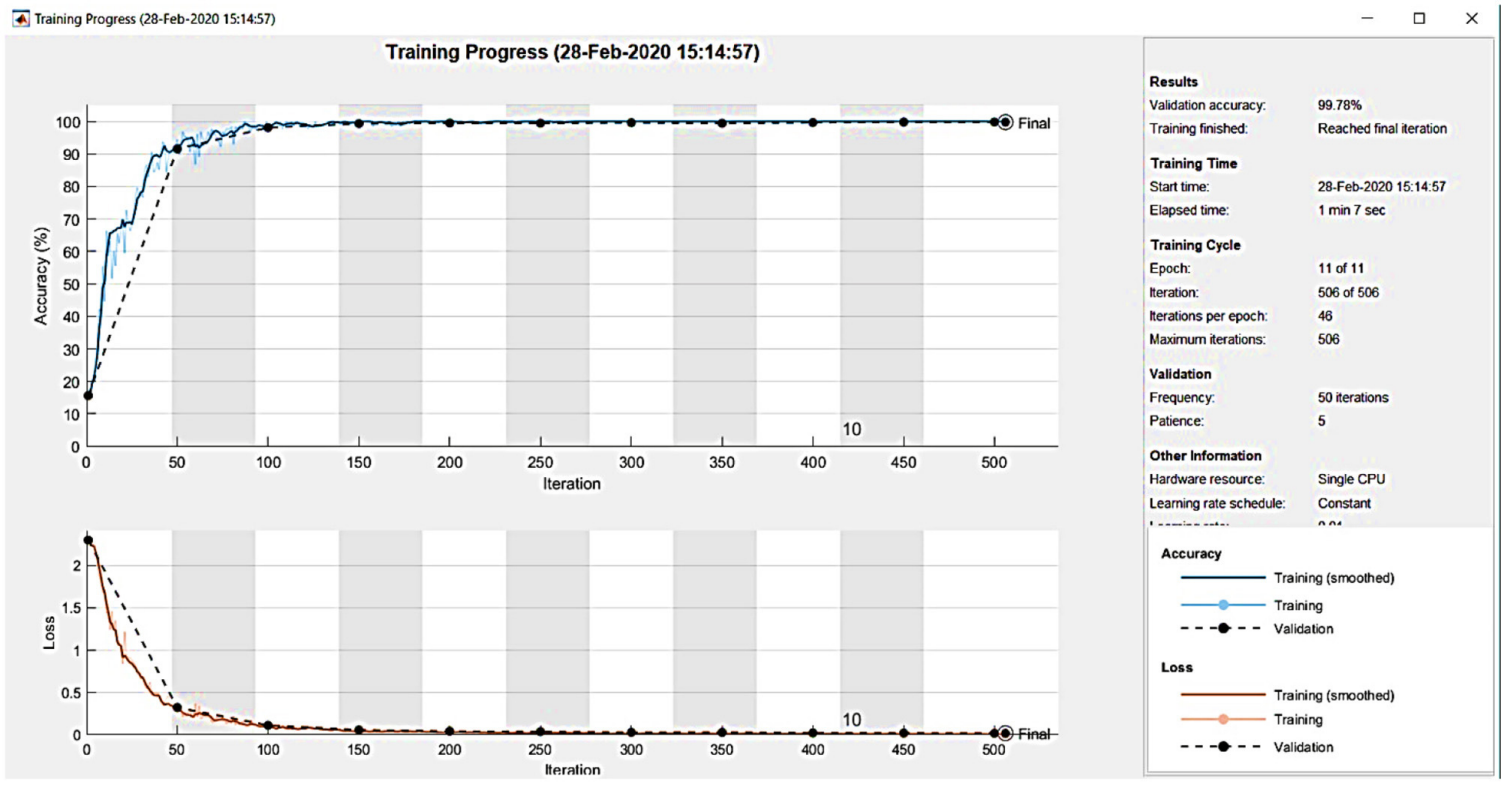
CNN training process.

**Figure 9:**
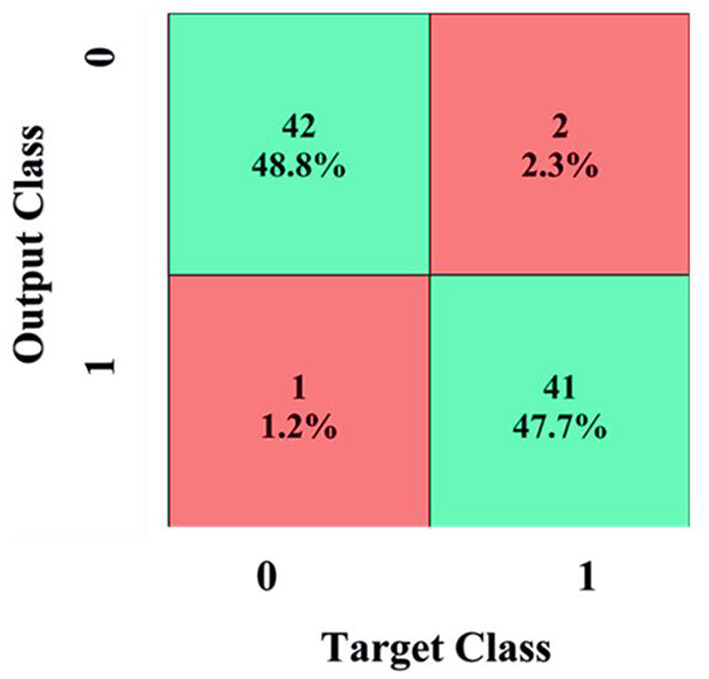
Confusion matrix.

Figure 8 shows the training plot of the CNN. The outcome of the proposed CAD system is demonstrated in the form of a confusion matrix as detailed in Figure 9. The total number of images is 855 including 635 NC and 220 AD patients.

To demonstrate the superior performance, the developed system was repeated five times and the mean values were reported. Efficacy of the developed system is evaluated by computing evaluation parameters which are given in section 2. The outcome of the proposed CAD system for the testing sample is demonstrated in the form of a confusion matrix as detailed in Figure 9.

**Figure 10:**
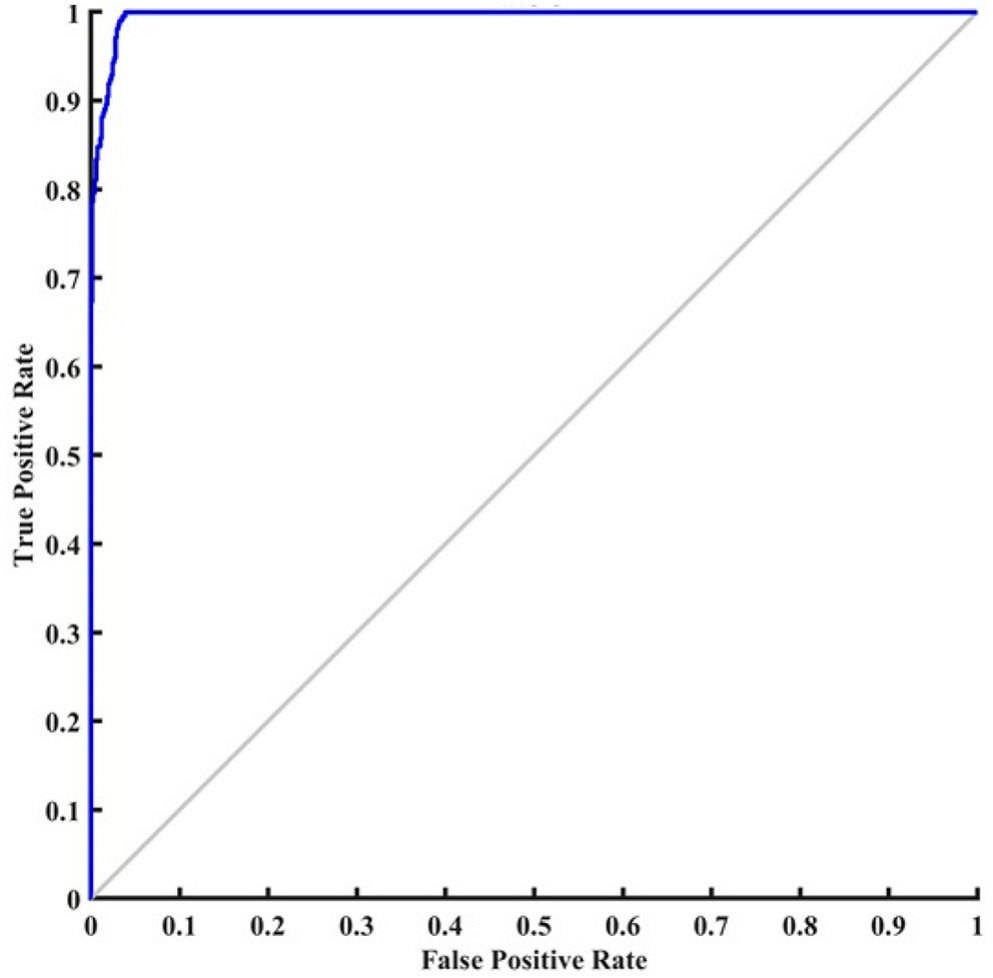
ROC curve of the proposed system.

In Figure 9, 42 images are correctly diagnosed as NC patients and 41 images are correctly classified as AD patients. A total of 2 images out 43 images are mistakenly identified as AD (2.3%). Similarly, one image is misdiagnosed as NC (1.2%). For NC patients, out of 43 patients, 48.8% are correctly diagnosed as NC and 2.3% are wrongly diagnosed. For AD patients, out of 43 patients, 47.7% are correctly classified as AD and 1.2% are wrongly classified. Overall, 96.5% of the photos are correctly classified, whereas 4.5% are incorrectly categorized. ROC is a mathematical tool used to assess the separation ability of the classification system. The true positive rate is compared against the false positive rate in a ROC chart. It is investigated by adjusting the threshold value. Figure 10 shows the ROC of the proposed system. The area under the curve (AUC) of the proposed system is 0.95.

### 5.4. Effect Analysis

In terms of accuracy, sensitivity, and specificity, this section compares the efficacy of the proposed CAD system to that of existing approaches. In particular, the experimental consequences reported in state-of-the-art methods such as Cabral and Silveria (4), Liu et al. (23), Islam and Zhang (24), Silveira and Marques (25), and Poongodi and Bose (26) are compared with the proposed system, as given in Table 3.

**Table 3:** Performance comparison of the proposed system with existing systems.

**Author**	**Modality**	**Method**	**Accuracy (%)**	**Sensitivity (%)**	**Specificity (%)**
Cabral and Silveria (4)	PET	PCA-SVM	74.18	92.75	15.91
Liu et al. (23)	FDG-PET	SVM	90	82.7	80.4
Islam and Zhang (24)	MRI	CNN	95.3	84.4	71.4
Silveira and Marques (25)	MRI	DNN	86.1	84	74.8
Poongodi and Bose (26)	FDG-PET	CNN-RNN	95.3	91.4	91
Proposed	FDG-PET	CNN	96.8	94	96

Cabral and Silveria (4) proposed a method to differentiate NC from AD using PET images. Input images were preprocessed using SPM. Feature vectors were extracted with PCA. SVM classifier discriminated NC from AD based on the feature vectors. Regional analysis-based classification system was proposed by Liu et al. (23). In this approach, the PET image was preprocessed using SPM. SVM classifier was used to carry out the classification process. Islam and Zhang (24) investigated the strength of CNN for diagnosing AD. They converted the 3D MRI images into 2D images. To increase the image's quality, a few preprocessing techniques, such as noise reduction, edge recognition, and segmentation were used. The preprocessed image was fed as an input to the CNN. The deep learning neural network provided a better result compared to the existing methods.

**Figure 11:**
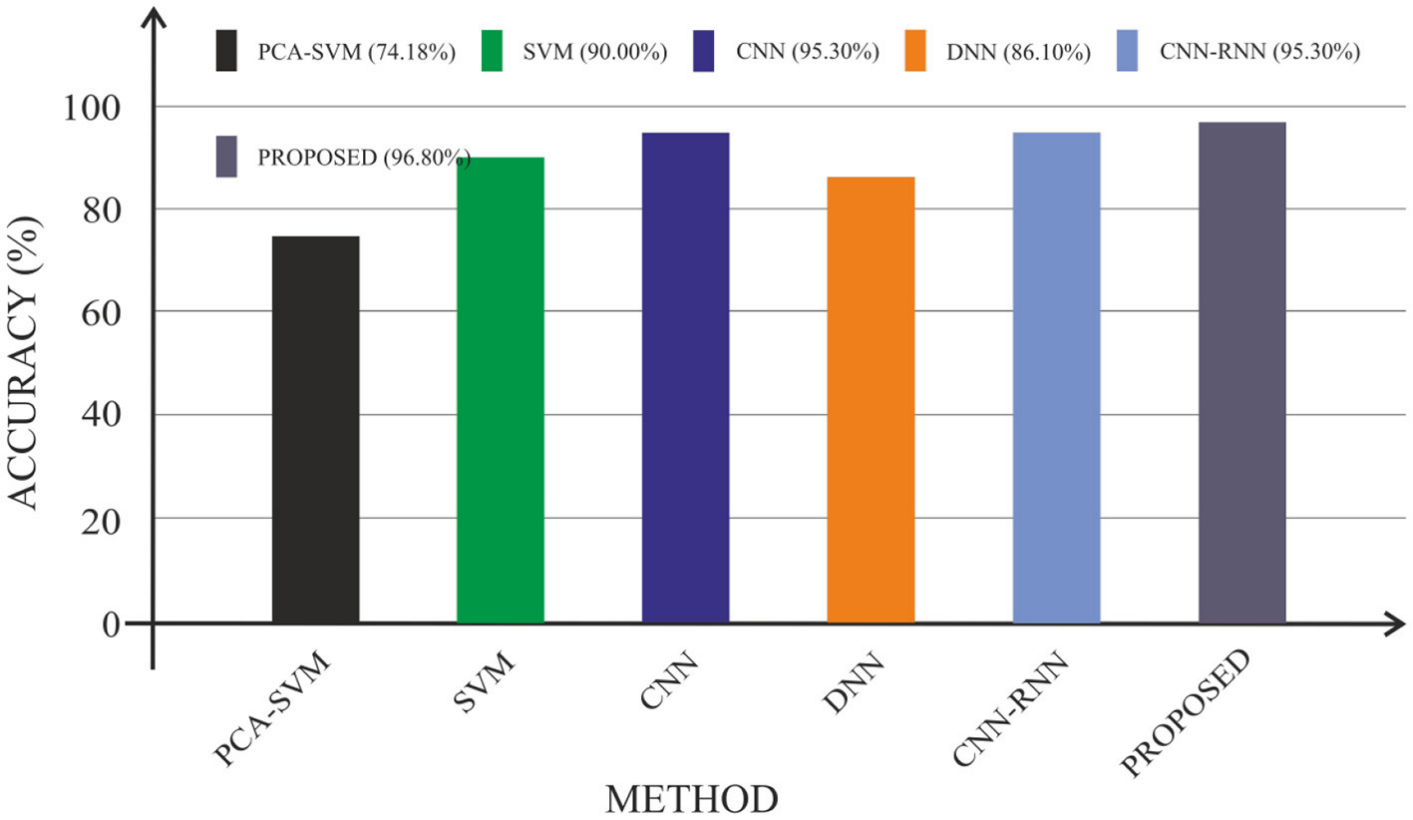
Performance comparisons in terms of accuracy.

Silveira and Marques (25) presented a classification system based on a CNN and extreme machine learning classifier. CNN was utilized to extract characteristics from MRI images in this system. Then, with the help of free surfer, the features were mined. Extreme machine learning classifier performed the classification task. The drawback of the system is high computational overhead. A hybrid method of CNN and RNN for AD diagnosis was presented by Poongodi and Bose (26). In this method, 3D PET images were transformed into 2D slices along axial, coronal, and sagittal directions to reduce the computation time. At regular intervals, the transformed slices were divided into a number of groups. The resultant groups were fed as input to the CNN. To extract inter slice features, RNN was used. Prediction score was calculated using the weighted averaging method. However, this method needs more memory and time. Figure 11 shows a graphic comparison of the suggested CAD system's classification with the current approaches.

## 6. Conclusion and Future Works

In this paper, a CAD device for discriminating AD from NC patients based on features from 18FDG-PET images was proposed and investigated properly. The proposed CAD system was designed by using a convolutional neural network. The FDG-PET images were decomposed into several 2D slices for extracting the features. Subsequently, the slices were grouped at some intervals without overlaps. The proposed CAD machine was confirmed on the ADNI dataset. The obtained simulation results showed that the proposed system provided exceptional performance when compared to the existing methods. Simulation results clearly demonstrated that the proposed CNN possessed higher potential to differentiate AD from NC with better classification accuracy and robustness. Future study will focus on improving the classification accuracy by extending the suggested CAD system to incorporate data from other sources. The overall performance of the suggested CAD system will be evaluated using a large number of samples. In addition, the potential of using different deep learning neural networks will be explored in a wider scenario.

## Data Availability Statement

The raw data supporting the conclusions of this article will be made available by the authors, without undue reservation.

## Author Contributions

AA: conceptualization, original draft writing, and implementation. PM and MH: funding acquisition. SB: supervision. All authors contributed to the article and approved the submitted version.

## Conflict of Interest

The authors declare that the research was conducted in the absence of any commercial or financial relationships that could be construed as a potential conflict of interest.

## Publisher's Note

All claims expressed in this article are solely those of the authors and do not necessarily represent those of their affiliated organizations, or those of the publisher, the editors and the reviewers. Any product that may be evaluated in this article, or claim that may be made by its manufacturer, is not guaranteed or endorsed by the publisher.

## References

[1] Wysoczański D Mroczka J Polak AG. Performance analysis of regularization algorithms used for image reconstruction in computed tomography. Bull Polish Acad Sci Techn Sci. (2013) 61:467–74. 10.2478/bpasts-2013-0046

[2] Poongodi M Sharma A Vijayakumar V Bhardwaj V Sharma AP Iqbal R. Prediction of the price of Ethereum blockchain cryptocurrency in an industrial finance system. Comput Elect Eng. (2020) 81:106527. 10.1016/j.compeleceng.2019.106527

[3] Grochowski M Kwasigroch A Mikołajczyk A. Selected technical issues of deep neural networks for image classification purposes. Bull Polish Acad Sci Tech Sci. (2019) 61:363–76. 10.24425/bpas.2019.128485

[4] Cabral C Silveria M. Classification of Alzheimer's disease from FDG-PET images using Favorite class ensemble. In: Proc. 35th International Conference of the IEEE EMBS Engineering in Medicine and Biology Society. Osaka (2013). p. 2477–80. 10.1109/EMBC.2013.6610042 24110229

[5] Association A. 2019 Alzheimer's disease facts and figures. Alzheimer's Dementia. (2019) 15:321–87. 10.1002/alz.12068 32157811

[6] Stasiak B Tarasiuk P Michalska I Tomczyk A. Application of convolutional neural networks with anatomical knowledge for brain MRI analysis in MS patients. Bull Polish Acad Sci Tech Sci. (2018) 66:857–68. 10.24425/bpas.2018.125933

[7] Illán IA Górriz IM Ramírez J Gonzalez DS. 18F-FDG PET imaging analysis for computer aided Alzheimer's diagnosis”. Inf Sci. (2011) 181:903–16. 10.3389/fnagi.2018.00158 21158320 PMC2994934

[8] Garali I Adel M Bourennane S Guedj E. Region based brain selection and classification on PET images for Alzheimer's disease computer aided diagnosis. In: Proc IEEE International Conf on Image Processing. Quebec City, QC (2015). p. 1473–7.

[9] Markiewicz T Swiderska-Chadaj Z Gallego J Bueno G Grala B Lorent M. Deep learning for damaged tissue detection and segmentation in Ki-67 brain tumor specimens based on the U-net model. Bull Polish Acad Sci Tech Sci. (2018) 66:849–56. 10.24425/BPAS.2018.125932

[10] Poongodi M Bose S. A firegroup mechanism to provide intrusion detection and prevention system against DDoS attack in collaborative clustered networks”. Int J Inf Security Privacy. (2014) 8:1–18. 10.4018/IJISP.2014040101

[11] Poongodi M Bose S. Stochastic model: reCAPTCHA controller based co-variance matrix analysis on frequency distribution using trust evaluation and re-eval by Aumann agreement theorem against DDoS attack in MANET.” *Clust Comput*. (2015) 18:1549–59. 10.1007/s10586-015-0496-y

[12] Gray KR Wolz R Keihaninejad S Heckemann RA Aljabar P Hammers A. Regional analysis of FDG-PET for use in the classification of Alzheimer's disease. In: Proc. IEEE International Symposium on Biomedical Imaging: From Nano to Macro. Chicago, IL (2011). p. 1082–5.

[13] Iwendi C Bashir AK Peshkar A Sujatha R Chatterjee JM Pasupuleti S. COVID-19 patient health prediction using boosted random forest algorithm. Front Publ Health. (2020) 8:357. 10.3389/fpubh.2020.00357 32719767 PMC7350612

[14] Kutia S Chauhdary SH Iwendi C Liu L Yong W Bashir AK. Socio-Technological factors affecting user's adoption of eHealth functionalities: A case study of China and Ukraine eHealth systems. IEEE Access. (2019) 7:90777–88. 10.1109/ACCESS.2019.292458427295638

[15] Iwendi C Moqurrab SA Anjum A Khan S Mohan S Srivastava G. N-sanitization: a semantic privacy-preserving framework for unstructured medical datasets. Comput Commun. (2020) 161:160–71. 10.1016/j.comcom.2020.07.032

[16] Patel H Singh Rajput D Thippa Reddy G Iwendi C Kashif Bashir A Jo O. A review on classification of imbalanced data for wireless sensor networks. Int J Distrib Sensor Netw. (2020) 16:1550147720916404. 10.1177/1550147720916404

[17] Lei B Liang E Yang M Yang P Zhou F Tan EL. Predicting clinical scores for Alzheimer's disease based on joint and deep learning. Exp Syst Appl. (2022) 187:115966. 10.1016/j.eswa.2021.11596630932861

[18] Lin E Lin CH Lane HY. Deep learning with neuroimaging and genomics in Alzheimer's disease. Int J Mol Sci. (2021) 22:7911. 10.3390/ijms22157911 34360676 PMC8347529

[19] Venugopalan J Tong L Hassanzadeh HR Wang MD. Multimodal deep learning models for early detection of Alzheimer's disease stage. Sci Rep. (2021) 11:1–13. 10.1038/s41598-020-74399-w 33547343 PMC7864942

[20] He Y Wu J Zhou L Chen Y Li F Qian H. Quantification of Cognitive Function in Alzheimer's Disease Based on Deep Learning. Front Neurosci. (2021) 15:178. 10.3389/fnins.2021.651920 33815051 PMC8010261

[21] Reddy T Bhattacharya S Maddikunta PKR Hakak S Khan WZ Bashir AK. Antlion re-sampling based deep neural network model for classification of imbalanced multimodal stroke dataset. Multimedia Tools Appl. (2020) 5:1–25. 10.1007/s11042-020-09988-y

[22] Srinivasu PN Bhoi AK Jhaveri RH Reddy GT Bilal M. Probabilistic deep Q network for real-time path planning in censorious robotic procedures using force sensors. J Real Time Image Process. (2021) 18:1773–85. 10.1007/s11554-021-01122-x

[23] Liu M Cheng D Yan W. Classification of Alzheimer's disease by combination of convolutional and recurrent neural networks using FDG-PET images. Neuro Inf. (2018) 12:35. 10.3389/fninf.2018.00035 29970996 PMC6018166

[24] Islam J Zhang Y. Brain MRI analysis for Alzheimer's disease diagnosis using an ensemble system of deep convolutional neural networks. Brain Inf Springer. (2018) 5:2. 10.1186/s40708-018-0080-3 29881892 PMC6170939

[25] Silveira M Marques J. Boosting Alzheimer disease diagnosis using PET images. In: Proc. 20th IEEE International Conference on Pattern Recognition (ICPR). Istanbul (2015). p. 2556–9.

[26] Poongodi M Bose S. Detection and Prevention system towards the truth of convergence on decision using Aumann agreement theorem. Proc Comput Sci. (2014) 50:244–51. 10.1016/j.procs.2015.04.053

